# Evaluating the Serum Levels of CD73 in Patients with Head and Neck Squamous Cell Carcinoma 

**DOI:** 10.30476/dentjods.2025.102855.2403

**Published:** 2026-06-01

**Authors:** Razieh Zare, Mohammad Ali Ranjbar, Ava Ebrahimi, Mahyar Malekzadeh, Bijan Khademi, Najmeh Savadi

**Affiliations:** 1 Oral and Dental Disease Research Center, Dept. of Oral and Maxillofacial Pathology, School of Dentistry, Shiraz University of Medical Sciences, Shiraz, Iran.; 2 Dept. of Oral and Maxillofacial Pathology, School of Dentistry, Shiraz University of Medical Sciences, Shiraz, Iran.; 3 Student’s Research Committee, Biomaterial Research Center, School of Dentistry, Shiraz University of Medical Sciences, Shiraz, Iran.; 4 Shiraz Institute for Cancer Research, School of Medicine, Shiraz University of Medical Sciences, Shiraz, Iran.; 5 Dept. of Otolaryngology, Khalili Hospital, Shiraz Institute for Cancer Research, School of Medicine, Shiraz University of Medical Sciences, Shiraz, Iran.; 6 Dept. of Dental Public Health, School of Dentistry, Bushehr University of Medical Sciences, Bushehr, Iran.

**Keywords:** CD73 antigen, Antigen expression, Head and neck carcinoma, Squamous cell carcinoma, Clinicopathological parameters/ characteristics

## Abstract

**Background:**

The expression of CD73 antigen is associated with different prognoses in different carcinomas. However, few studies have assessed the level of this marker in squamous cell carcinoma (SCC) as the most common malignancy of the head and neck region.

**Purpose:**

The aim of this study was to determine the serum levels of CD73 in patients with head and neck SCC to explain their association with clinicopathological parameters.

**Materials and Method:**

This cross-sectional study was done on 60 samples taken from patients with head and neck SCC. A total of 28 healthy individuals were enrolled as the control group. Using the sandwich ELISA method, the serum levels of CD73 were measured. The data were statistically analyzed using the independent t-test and the Kruskal-Wallis test.

**Results:**

The mean serum level of CD73 in the SCC patients (114.38 ng/ml, n=60) was almost similar to that of the healthy controls (137.22 ng/ml, n=28; *p* Value=0.098). The mean serum levels of CD73 were greater in patients with III/IV-stage tumors than in those with I/II-stage tumors (121.37±55.96 ng/ml, n=28 vs 108.27±39.44 ng/ml, n=32; *p*= 0.294). No correlation was found between the serum levels of CD73 and sex, age, tumor size, lymph node metastasis, and other organ metastasis.

**Conclusion:**

The results of the current study revealed no significant relationship between the serum levels of CD73 and the clinicopathological factors in patients with head and neck SCC. These results suggest that the serum levels of CD73 may not be a useful biomarker for the recognition of the clinical behavior of head and neck SCC. However, the actual role of CD73 in SCC remains unclear and requires further research.

## Introduction

Squamous cell carcinomas (SCCs) have been reported as the most common malignancies of the head and neck region [ 1 ]. Accounting for at least 90% of all head and neck cancers, these highly aggressive neoplasms affect more than half a million patients worldwide each year [ 2 - 3 ]. According to GLOBOCAN (global cancer statistics, 2018), more than 800,000 new cases of head and neck SCC are diagnosed annually [ 4 ]. 

A multifactorial etiology is considered for SCC. Both extrinsic factors (including smoking, alcohol consumption, and viral infections) and intrinsic factors (such as genetic factors, immunosuppression, and chronic iron deficiency anemia) are the proposed risk factors for SCC [ 5 - 6 ]. Like other carcinomas, more cases of this kind of cancer have been reported in older age and in male individuals [ 7 ]. However, in recent years, lifestyle changes have reversed this trend with increased incidence in younger age and in women [ 6 ]. 

SCC is one of the most lethal types of head and neck cancers, with a 5-year survival rate of around 40-50% [ 3 ]. Only about one-fourth of the patients are diagnosed at the early stages of the disease the majority of them show advanced stages at the time of diagnosis [ 8 ]. Since diagnosis at late stages has been found to be strongly associated with worse survival for all head and neck SCC patients [ 8 ], an urgent need is felt for identifying the specific biomarkers that can be used to improve cancer prognosis. 

Cluster of differentiation 73 (CD73) is one of the cell surface molecules widely expressed on multiple cells (especially on T regulatory cells) [ 9 - 10 ]. This molecule acts as a rate-limiting enzyme and usually catalyzes the dephosphorylation pathway of extracellular adenosine monophosphate to adenosine [ 11 - 12 ]. The adenosinergic pathway leads to the immune system suppression and has a critical role in maintaining immune system homeostasis [ 13 ]. In addition, recent studies have reported that this immune-inhibitory pathway (CD73-adenosine) is involved in the formation of an immunosuppressive microenvironment in different cancers [ 12 ]. 

Several studies have revealed that the CD73 antigen is expressed highly in different carcinomas such as pancreatic, prostate, ovarian, bladder, breast, and gastric cancers [ 10 , 14 - 18 ]. Some articles have demonstrated that a high CD73 expression is associated with poor cancer prognosis, characterized by promoting cancer cell aggressiveness, angiogenesis, and metastasis [ 18 - 20 ]. However, some researchers have reported that a high CD73 expression is associated with a favorable prognosis in ovarian cancer [ 15 , 21 ]. So far, few studies have assessed the level of this marker in SCC by immunohistochemical (IHC) evaluation [ 16 , 19 ]. Therefore, the aim of this study was to evaluate the serum levels of CD73 in head and neck SCC and to explain their associations with clinicopathological characteristics by using the sandwich enzyme-linked immunosorbent assay (ELISA). 

## Materials and Method

This retrospective cross-sectional study was conducted on the documents and serum samples of patients who were hospitalized in the ENT unit of Shiraz Khalili Hospital, Shiraz, Iran. The study protocol was approved by the Ethics Committee of Shiraz University of Medical Sciences (Ethical code: IR.Sums.Dental.REC.1397. 57). 

The serum samples of 60 patients with head and neck SCC whose disease had been diagnosed by histopathological methods were evaluated. The samples with sufficient amounts and explicit diagnosis of SCC were included in the study, and those with an uncertain diagnosis were excluded. The documents and serum samples of twenty-eight healthy individuals were also chosen to be assessed as the control group. Both the patient and control groups were matched based on sex and age.

### Serum analysis

After obtaining written informed consent from the participants, blood samples were taken from the patient and control groups. To obtain serum, the blood samples were centrifuged for 10 minutes at 1000 RPM and were subsequently kept in a deep freezer at -80°C until determination. The serum levels of CD73 were measured using the ELISA kit according to the manufacturer’s instructions (Catalog Number D3300, R&D Systems, USA). 

### Statistical analysis

The SPSS software (version 16) was employed for the statistical analysis. Using the independent t-test, the CD73 serum levels of the patient and control groups were compared. Afterwards, the Kruskal-Wallis test was used to define the relationship between the serum levels of CD73 and the clinical data. The differences were considered statistically significant for *p*< 0.05. 

## Results

Considering the inclusion and exclusion criteria of study, a total of 60 patients with head and neck SCC and 28 healthy controls were enrolled in the study. Table 1 shows the clinical data as well as the CD73 serum levels of the individuals who were evaluated. Among the 88 individuals, 23 (26.1%) were men and 65 (73.9%) were women. The average ages of the females in the patient and control groups were 65.33±13.99 and 64.25±10.26, respectively. The males also had matching average ages in both patient and control groups (60.82±12.72 and 59.90±14.49, respectively). No significant differences were seen between the CD73 serum levels and the participants’ gender or age. 

**Table 1:** The correlations between the serum levels of CD73 and the clinical parameters in the study groups

Groups	Gender (female/male)	Age (mean±SD)	Mean CD73 concentration±SD	Metastasis to other organs (+: -)	Lymph node metastasis (+: -)	Tumor stage (number of patients)				Tumor size (number of patients)		
						1	2	3	4	1	2	3
Control	8:20	61.14±13.38	137.223 ± 63.52	-	-	-	-	-	-	-	-	-
SCC	15:45	61.32±13.28	114.386 ± 47.89	6:54	13:47	13	19	15	13	14	30	16
Total	23:65	61.69±13.10	-	6:54	13:47	13	19	15	13	14	30	16
*p* Value*	0.722	0.955	0.098	0.466	0.330	0.4				0.074		

* T-test and Kruskal Wallis test

The mean serum levels of CD73 in the patients with head and neck SCC (114.38 ng/ml, n=60) were almost similar to those of healthy individuals (137.22 ng/ml, n=28; *p*= 0.098). The serum levels of CD73 were greater in patients with III/IV-stage tumors than in those with I/II-stage tumors (121.37±55.96 ng/ml, n=28 vs 108.27 ±39.44 ng/ml, n=32; *p*= 0.294).

No significant correlation was found between the CD73 serum levels and sex, age, tumor size, lymph node metastasis, and other organ metastasis. 

## Discussion

As far as we know, the current research is one of the few studies that evaluated the role of CD73 serum levels in patients with head and neck SCC. The present study revealed similar results with respect to the serum levels of CD73 in both the patient and control groups. The analysis of various parameters, including tumor stage, tumor size, and lymph node metastasis, and so on, revealed no significant relationship between the serum levels of CD73 and the mentioned parameters. Many studies have reported that CD73 is overexpressed in many cancers and plays an important role in carcinogenesis. Several studies have investigated the expression levels of CD73 in tumor and normal tissues [ 10 , 14 - 18 ]. 

For instance, a systematic review and meta-analysis [ 10 ] evaluated the expression of CD73 comprehensively in distinct types of cancers. They evaluated some types of tumors like liver cancer, lung SCC, and colorectal cancer, and these tumors showed CD73 expression levels similar to those in matched normal tissues. However, this review showed that remarkably higher CD73 expression levels were observed in tumor tissues compared to normal tissues in most kinds of cancer; except for cecum adenocarcinoma and ovarian cancer which had markedly lower CD73 expression levels than those in matched normal tissues [ 10 ]. In the current study, the CD73 serum levels were also similar in the patient and healthy groups. 

To understand whether CD73 could be considered as a serologic prognostic biomarker or not, many articles have analyzed the correlation between the expression of CD73 and the clinicopathological characteristics of different tumors [ 19 - 24 ]. Tumor size, tumor clinical stage, invasion, and metastasis to lymph nodes were some of the characteristics examined in these studies. In the present study, no significant relationship was observed between the serum levels of CD73 and the above-mentioned clinicopathological factors. Similarly, a previous study conducted on patients with breast carcinoma, has also reported no significant correlation between the CD73 expression levels and tumor grading, tumor size, lymph node metastasis, and tumor histologic type [ 22 ].

In contrast to the results of the current study, some studies have found a statistically significant relationship between the expression levels of CD73 and the clinical factors in patients with ovarian [ 23 ], gastric [ 20 ], and prostate [ 14 ] cancers. A recent study of patients with oral SCC [ 19 ] has also reported a significant association between the overexpression of CD73 and tumor clinical stage, degree of differentiation, tumor size, and lymph node metastasis. The findings of these studies suggested that the overexpression of CD73 may be a novel marker of poor prognosis and adverse clinical outcomes in cancer patients. 

However, research conducted on patients with epithelial ovarian cancer revealed that the overexpression of CD73 was more commonly observed in patients with lower stage, better differentiation, and no lymphovascular involvement. Hence, this study reported CD73 as a potential indicator of good prognosis [ 15 ]. Gao *et al*. [ 21 ] stated that the propagation effect of CD73 and the preventive effect of adenosine on the growth and migration of cervical cancer cells seemed contradictory. They proved that the propagation effect of the overexpression of CD73 on the growth and migration of *in vitro* cells was related to its enzymatic task [ 21 ]. They also mentioned that the adenosine concentration should be > 100 µm to have a preventive effect [ 21 ].

In the present study, the serum levels of CD73 in the samples were measured using the sandwich ELISA method, while some previous studies used IHC analysis to assess the expression levels of CD73 in cancer tissues [ 19 - 20 , 22 - 23 ]. According to recent evidence, the serum levels of CD73 possibly increase in cancer patients as a result of tissue-associated inflammation/ hypoxia. This probably reflects the elevated expression of CD73 within the tumor microenvironment [ 24 ]. If the actual role of CD73 in the progression of head and neck SCC is verified, then this marker can be promising for future treatment strategies. As the serum levels of CD73 can be measured quickly and easily in cancer patients, therapies targeting the CD73/adenosine pathway might be employed for their treatment.

The retrospective nature of the present research and the limited number of participants could be considered as the limitations of this study. To obtain better results, future studies are recommended with a prospective nature and larger sample sizes. 

Additional and combined molecular studies with other markers in a larger sample group are required to confirm the actual role of CD73 in the progression of head and neck SCC.

## Conclusion

The findings of the current study demonstrated no significant relationship between the serum levels of CD73 and the clinicopathological factors in patients with head and neck SCC. The serum levels of CD73 were almost similar in patients with SCC and healthy controls. The results of the present study suggested that CD73 could not be considered a good marker for the recognition of the clinical characteristics of head and neck SCC. Therefore, more studies are required for a better understanding of the real role of CD73 in patients with head and neck SCC. 
